# Supplementary material for: Quantization of geometric-like coupling in gravitational field based on characterization and transformation
Source: Sci Rep. 2026 Jul 2;16:20781. doi: 10.1038/s41598-026-53091-5 (PMC13338383; doi:10.1038/s41598-026-53091-5)
Supplement: Supplementary file 1 — Supplementary Information. [file 41598_2026_53091_MOESM1_ESM.docx]

**Quantization of geometric-like coupling in gravitational field based on characterization and transform**

**Chen Yang**^[[1]](#footnote-1)^

^1^Faculty of Science and Engineering, The University of Manchester, Manchester, M13 9PL, UK

ABSTRACT

Connections between the formulations of physics and geometry have been evident throughout history, from classical mechanics to general relativity. Independently, quantum mechanics has been established in flat space. In this study, we investigate the geometric-like coupling of a test particle and its quantized form in a gravitational field. The main text consists of three parts: the characterization of the particle and its operator form is pointwise established. Minimal coupling with the electromagnetic potential is analyzed as a reference via an infinitesimal transform. Subsequently, the geometric coupling from the geodesic strain and its associated phase transform of the initial parallel test particle is formally studied. Within the gravitational field equation, the phase transform is specified via metric tensors in general. The linearized field condition is analyzed explicitly to show the local analogy between the four-potential in gauge-like and geometric-like couplings. From the isomorphism and function maps, the amplitude and phase in the operator form are translated to the quantum form and the Schrödinger-like equation is obtained. The representation of local equivalence and the phase transform condition is formulated. Examples of the phase shift and potential well, as well as the geometric Aharonov–Bohm effect, are studied for potential applications. Finally, the geometric-like and gauge-like couplings are summarized based on the concept of characterization and transform.

# Introduction

Historically, the interplay between classical mechanics and differential geometry has gradually emerged from studies on the trajectories of particles under conservative and nonconservative potentials [1, 2]. In addition to flat space, geodesics in configuration spaces (for example, Hertz’s forceless mechanism) have motivated the geometric formulation of classical mechanics and field theories [3, 4]. Continuous studies have contributed to the development of general relativity, which describes the gravitational potential in Riemann geometry [5, 6]. Independently, the wavefunctions and dynamic operators of particles in quantum mechanics have been established in flat space [7]. Later, in electrodynamics, the interaction between the particle and field is formulated from the coupling between the quantum operator and field potentials under gauge invariance. [8]. However, the geometric formulation of gravitational potentials is distinct from conventional gauge potential fields [9, 10].

Owing to the geometric nature of the gravitational potential, many attempts have been made to bridge the geometric formulation with the conventional gauge potential formulation [11 - 13]. The reported studies have provided many important discoveries (for example, gauge treatment, Schrödinger-Newton equations, background field, loop quantum gravity, and effective action) that remain active in the academic field [14 - 23]. Semi-classical gravity has been one of the above attempts where the geometric feature of the gravitational field is considered in classical form [24, 25]. Recently, the study of geometric feature of quantum evolution bridge the semi-classical gravity with geometric phase that arise in other branches such as condense matter physics [26 - 27]. Alternatively, parallel explorations of alternative or extended forms of general relativity have been reported in the literature [29]. Studies on the extended theories of gravity have been reported by several authors in multiple directions, including quantized gravity and large-scale structures in cosmography [30 - 33]. Comprehensive views of the relevant directions have been reported in the literature [34]. A recent study on quantum gravity at low energy scales has been reported in [35]. Despite studies on the connection between the gravity field and quantum mechanics, the primary aim is to arrive at both subjects with a certain consistency. Simultaneously, there are remaining open questions on different methods during the connection of the two important theories from conceptual and technical (formulation) aspects, as mentioned in the literature [36 - 44]. A comprehensive summary of the different methods and questions in the recent progress of quantum gravity is given in [45, 46].

In this manuscript, we study the geometric coupling on test particles based on their pointwise characterization and infinitesimal transform. To start with, the pointwise characterizations of test particles and their matrix transform are introduced. By evaluating the identical map on momentum along the geodesics, the operator form is established as the basis for evaluating the matrix transform in gauge-like and geometric-like couplings. In the second part, coordinate deformation (map) owing to geodesic deviation (strain) and their associated phase transform are investigated. From the inner product of the phase, the coordinate variation induced an equivalent momentum variation that fulfilled phase invariance. With the governing equation of field tensors, the geometric coupling is configured as a function that depends on the variation of metric tensors. Under the linearized field condition, the geometric coupling becomes an analogous form to electromagnetism, which is locally coupled with test particles. In the third section, by observing the isomorphism between the operator form and quantum form, function maps are introduced to translate (time-independent) amplitude and phase to their quantum counterparts. Some examples are studied to cover the principle of equivalence from both magnitude and phase aspects. The phase shift and finite potential well are analyzed to bring discretized features of the geometric strain. The geometric Aharonov–Bohm effect is studied from the above results and linked to experimental studies in the literature. Finally, we summarize the geometric-like and gauge-like couplings in terms of characterization and matrix transform.

# Results

## Operator of particle and gauge

In this section, we studied the operator form and its transform in inertial and curvilinear frames. By considering the particle or field as pointwise characterizations, we studied their transforms via infinitesimal transforms and established the operator form. As an example, the minimal coupling from electromagnetism is presented as the transform between momentum and gauge potential.

### ***Characterizations of particle and field***

$\left\{ r_{i},p_{i},S \right\}$From classical mechanics in curvilinear coordinate of a test particle [1], t$O$he local inertia frame can be specified by

| $\boldsymbol{r}_{i}\boldsymbol{=}\left[ \boldsymbol{r}_{0},\boldsymbol{r}_{1},\boldsymbol{r}_{2},\boldsymbol{r}_{3} \right] ;\boldsymbol{r}=\left[ \boldsymbol{r}_{1},\boldsymbol{r}_{2},\boldsymbol{r}_{3} \right]$ | (1) |
| --- | --- |

and

| $\boldsymbol{r}_{0}(t)\boldsymbol{=-v}t ;\boldsymbol{v}=\frac{d\boldsymbol{r}}{dt} ,$ | (2) |
| --- | --- |

where $r_{i}$ is the four-displacement (position), $\boldsymbol{r}$ refers to displacement and $t$ refers to time, $\boldsymbol{v}$is the velocity as in Cartesian notation. The dynamic variables and action are connected via partial differential in local inertia frame,

| $p_{i}=\partial_{r_{i}}S=p-\frac{1}{v}H ,$ | (3) |
| --- | --- |

where $p$ refers to the momentum, $H$ refers to Hamiltonian and $p_{i}$ refers to four-momentum. By considering the evolution of particle as pointwise characterizations via above variables as below,

| $C=\left[ r_{i},p_{i},S \right] ,$ | (4) |
| --- | --- |

where $C$ contains four-vectors $r_{i},p_{i}$ and scalar $S$ and it is multi-dimension ($2n+1$) vector-like object compared to four-vector ($n)$. As in linear transform (mapping) [47], the variation of the pointwise characterization is represented via the matrix-like transform $\left[ T \right]$ between $C\to C^{'}$ as below,

| $\left[ r_{i},p_{i},S \right]\underset{\to}{\left[ T \right]}\left[ r_{i}^{'},p_{i}^{'},S^{'} \right] ; \left[ \begin{matrix} r_{i}^{'} \\ p_{i}^{'} \\ S^{'} \end{matrix} \right]=\left[ \begin{matrix} I_{ii^{'}}+\epsilon_{ii^{'}} & & \\ & I_{ii^{'}}+\sigma_{ii^{'}} & \\ & & I+\epsilon\end{matrix} \right]\left[ \begin{matrix} r_{i} \\ p_{i} \\ S \end{matrix} \right] ,$ | (5) |
| --- | --- |

where $\left[ T \right]$ is a diagonal-like matrix contain sub-matrices and $r_{i}^{'}=r_{i}+\Delta r_{i}$. In the above transform, $\left[ I_{ii^{'}} \right]$ is the identity matrix, $\left[ \epsilon_{ii^{'}} \right]$ denotes infinitesimal transform on coordinates, $\left[ \sigma_{ii^{'}} \right]$ denotes infinitesimal transform on momentum and $\epsilon$ refers to small parameter in exponential map. From literature, the classical electromagnetism in gauge and field potential form is given by coordinates $r_{i}$, four-potential $A_{i}$ and gauge $\chi$. By considering the evolution of the local field as pointwise characterizations with above variables, it gives a similar vector-like object $D=\left\{ r_{i},A_{i},\chi\right\}$ with the arbitrary small variation in the matrix transform,

| $\left[ r_{i},A_{i},\chi\right]\underset{\to}{[T]}\left[ r_{i}^{'},A_{i}^{'},\chi^{'} \right] ,$ | (6) |
| --- | --- |

where the potential variables and gauge are connected as below,

| $A_{i}=\partial_{r_{i}}\chi=A-\frac{1}{c}V ,$ | (7) |
| --- | --- |

where$c$ is the vacuum speed of light, $A$ refers to the vector potential, $V$ refers to scalar potential. In matrix form, the local modification of momentum $p_{i}$ via potential $A_{i}$is given by the relation below,

| $r_{i}^{'}=\left[ I_{ii^{'}} \right]r_{i} ;p_{i}^{'}=\left[ I_{ii^{'}}+\sigma_{ii^{'}} \right]p_{i} ;\left[ \sigma_{ii^{'}} \right]p_{i}=\left[ q_{ii^{'}} \right]A_{i} ,$ | (8) |
| --- | --- |

where $\left[ q_{ii^{'}} \right]$ denotes charge-like matrix. In matrix form, the local modification of momentum via potential is given by below relation,

| $r_{i}^{'}=\left[ I_{ii^{'}}+\epsilon_{ii^{'}} \right]r_{i} ; p_{i}^{'}=\left[ I_{ii^{'}} \right]p_{i}$ | (9) |
| --- | --- |

and

| $r_{i}^{'}=\left[ I_{ii^{'}}+\epsilon_{ii^{'}} \right]r_{i} ; A_{i}^{'}=\left[ I_{ii^{'}} \right]A_{i} .$ | (10) |
| --- | --- |

Next, we will explore the operator form of particle along geodesic and study their infinitesimal transform.

### ***Operator form and gauge potential***

By evaluating the dynamic variables along geodesics for particle, the following conditions can be found from Eq. (9),

| $p_{i}^{'}=\left[ I_{ii^{'}} \right]p_{i}\Rightarrow p_{i}^{'}\left( x_{a},\dot{x}_{a} \right)-p_{i}\left( x_{a},\dot{x}_{a} \right)=0 p_{i}^{'}-p_{i}=0, .$ | (11) |
| --- | --- |

and

| $\ddot{x}_{a}=\frac{d\dot{x}_{a}}{d\lambda}=\frac{d^{2}x_{a}}{d\lambda^{2}}+\Gamma_{bc}^{a}\frac{dx_{b}}{d\lambda}\frac{dx_{c}}{d\lambda}=0 .$ | (12) |
| --- | --- |

where $x_{a}\left( \lambda\right)$denotes four-displacement$x_{j}\left( \lambda\right)$, $\dot{x}_{a}\left( \lambda\right)$ denotes four-velocity$\dot{x}_{j}\left( \lambda\right)$ and $\lambda$ denotes scalar parametric time$\lambda$. By expanding the relation in Eq. (11) locally with respect to $x_{a}$ and $\dot{x}_{a}$ it gives,

| $p_{i}^{'}\left( x_{a},\dot{x}_{a} \right)-p_{i}\left( x_{a},\dot{x}_{a} \right)=p_{i}^{'}\left( x_{j},\dot{x}_{j} \right)-p_{i}\left( x_{j},\dot{x}_{j} \right)=0\Rightarrow\nabla_{x_{a}x}\boldsymbol{p}_{i}^{'}\dot{x}_{a}\dot{x}_{j}\boldsymbol{+}\nabla_{{\dot{\boldsymbol{x}}}_{ja}}\boldsymbol{p}_{i}^{'}{\ddot{\boldsymbol{x}}}_{ja}-\nabla_{x_{a}}\nabla_{x}\boldsymbol{p}_{i}\dot{x}_{a}\dot{x}_{j}\boldsymbol{-}\nabla_{\dot{x}_{a}}\nabla_{\dot{x}}\boldsymbol{p}_{i}{\ddot{\boldsymbol{x}}}_{ja}\boldsymbol{=}(\nabla_{x_{a}}\nabla_{x}\boldsymbol{p}_{i}^{'}\boldsymbol{-}\nabla_{x_{a}}\nabla_{x}\boldsymbol{p}_{i})\dot{x}_{a}\dot{x}_{j}+(\nabla_{\dot{x}_{a}}\nabla_{\dot{x}}\boldsymbol{p}_{i}^{'}-\nabla_{\dot{x}_{a}}\nabla_{\dot{x}}\boldsymbol{p}_{i}{)\ddot{\boldsymbol{x}}}_{ja}=0$ | (13) |
| --- | --- |


$\dot{x}_{j}$

By applying Eq. (12), the second bracket of Eq. (13) is cancelled and by expanding the first and second terms in the first bracket of Eq. (13), it leads to the following,

| $\nabla_{x_{a}}\nabla_{x}\boldsymbol{p}_{i}^{'}=\nabla_{x_{a}}\partial_{r_{i}^{'}}S^{'}=\nabla_{x}\frac{\partial}{\partial r_{i}^{'}}S^{'}=\partial_{r_{i}^{'}}\frac{\partial}{\partial r_{i}^{'}}\nabla_{x_{a}}\nabla_{x}S^{'}=\partial_{r_{i}^{'}}^{2}\frac{\partial^{2}}{\partial{r_{i}^{'}}^{2}}S^{'}\nabla_{x_{a}}\nabla_{x}\boldsymbol{r}_{i}^{'}=\left( \partial_{r^{'}}^{2}\frac{\partial^{2}}{\partial{r^{'}}^{2}}-\frac{1}{{\boldsymbol{v}^{\boldsymbol{'}}}^{2}}\partial_{t^{'}}^{2}\frac{\partial^{2}}{\partial{t^{'}}^{2}} \right)S^{'}\nabla_{x_{a}}\nabla_{x}\boldsymbol{r}_{i}^{'}$ | (14) |
| --- | --- |

and

| $\nabla_{x_{a}}\nabla_{x}\boldsymbol{p}_{i}=\nabla_{x_{a}}=\nabla_{x}\frac{\partial}{\partial r_{i}}S\partial_{r_{i}}S=\partial_{r_{i}}\frac{\partial}{\partial r_{i}}\nabla_{x_{a}}\nabla_{x}S=\partial_{r_{i}}^{2}\frac{\partial^{2}}{\partial r_{i}^{2}}S\nabla_{x_{a}}\nabla_{x}\boldsymbol{r}_{i}=\left( \partial_{r}^{2}\frac{\partial^{2}}{\partial r^{2}}-\frac{1}{\boldsymbol{v}^{2}}\partial_{t}^{2}\frac{\partial^{2}}{\partial t^{2}} \right)S\nabla_{x_{a}}\nabla_{x}\boldsymbol{r}_{i} ,$ | (15) |
| --- | --- |

and noting the second-order mixed derivatives can be cancelled from below condition,

| $\left( \partial_{r^{'}}\partial_{t^{'}}-\partial_{t^{'}}\partial_{r^{'}} \right)\left( \frac{\partial}{\partial r^{'}}\frac{\partial}{\partial t^{'}}-\frac{\partial}{\partial t^{'}}\frac{\partial}{\partial r^{'}} \right)S^{'}=0 ; \left( \partial_{r}\partial_{t}-\partial_{t}\partial_{r} \right)\left( \frac{\partial}{\partial r^{'}}\frac{\partial}{\partial t^{'}}-\frac{\partial}{\partial t^{'}}\frac{\partial}{\partial r^{'}} \right)S=0 .$ | (16) |
| --- | --- |

By substituting Eq. (14) and Eq. (15) into Eq. (11), and it leads to following relation at nearby points,

| $\left( \partial_{r^{'}}^{2}-\frac{1}{{v^{'}}^{2}}\partial_{t^{'}}^{2}\frac{\partial^{2}}{\partial{r^{'}}^{2}}-\frac{1}{{v^{'}}^{2}}\frac{\partial^{2}}{\partial{t^{'}}^{2}} \right)S^{'}\nabla_{x_{a}}\nabla_{x}\boldsymbol{r}_{i}^{'}-\left( \partial_{r}^{2}-\frac{1}{v^{2}}\partial_{t}^{2}\frac{\partial^{2}}{\partial r^{2}}-\frac{1}{v^{2}}\frac{\partial^{2}}{\partial t^{2}} \right)S\nabla_{x_{a}}\nabla_{x}\boldsymbol{r}_{i}=0 .$ | (17) |
| --- | --- |

$\left\{ r_{i},p_{i},S \right\}$Based on Eq. (17) we further study its solution on actions and dynamical variables. As the above covariant derivatives $\nabla_{x_{a}}\nabla_{x}\boldsymbol{r}_{i}$and$\nabla_{x_{a}}\nabla_{x}\boldsymbol{r}_{i}^{'}$ can be arbitrary, therefore Eq. (17) implies the following set of action wave equations in each local inertia frame,

| $\left( \partial_{r}^{2}-\frac{1}{v^{2}}\partial_{t}^{2}\frac{\partial^{2}}{\partial r^{2}}-\frac{1}{v^{2}}\frac{\partial^{2}}{\partial t^{2}} \right)S=0 ; \left( \partial_{r^{'}}^{2}-\frac{1}{{v^{'}}^{2}}\partial_{t^{'}}^{2}\frac{\partial^{2}}{\partial{r^{'}}^{2}}-\frac{1}{{v^{'}}^{2}}\frac{\partial^{2}}{\partial{t^{'}}^{2}} \right)S^{'}=0 .$ | (18) |
| --- | --- |

From basis solution of Eq. (13) is given by the plane waves form of action and superimposition,

| $S(r_{i})=-i\bar{S}\phi(\boldsymbol{r}_{i}) ; S^{'}\left( r_{i}^{'} \right)=-i\bar{S}\phi^{'}\left( \boldsymbol{r}_{i}^{'} \right) ,$ | (19) |
| --- | --- |

where $\bar{S}$ denotes the amplitude (time-independent) and $\phi$ denotes the (complex) phase function. From Eq. (3) and Eq. (5), the dynamical variables can be represented as below operator form,

| $H\left( r_{i} \right)=i\bar{S}\partial_{t}\frac{\partial}{\partial t}\phi; \boldsymbol{p}\left( r_{i} \right)=-i\bar{S}\partial_{r}\frac{\partial}{\partial r}\phi\Rightarrow p_{i}=-i\bar{S}\partial_{r_{i}}\phi=\bar{p}_{i}\phi=\bar{S}k_{i}\phi;H^{'}(r_{i})=i\bar{S}\frac{\partial}{\partial t^{'}}\phi^{'} ; p^{'}(r_{i})=-i\bar{S}\frac{\partial}{\partial r^{'}}\phi^{'} .$ | (20) |
| --- | --- |

where $k_{i}$ is the wavevector. Since the evolution from $r_{i}$ to $r_{i}^{'}$ is arbitrary, the operator form is established pointwise along the geodesic line. Based on similar condition on four-potential in Eq. (11), $A_{i}^{'}-A_{i}=0$, one could arrive the following set of gauge wave equations in each local inertia frame,

| $\left( \partial_{r}^{2}-\frac{1}{c^{2}}\partial_{t}^{2} \right)\chi=0 ; \left( \partial_{r^{'}}^{2}-\frac{1}{{c^{'}}^{2}}\partial_{t^{'}}^{2} \right)\chi^{'}=0 ,$ | (21) |
| --- | --- |

where$c=c^{'}$ is the vacuum speed of light. It is noticed that this is the pointwise version of Lorenze gauge condition in classical electromagnetism. The pointwise analogous on operator forms of particle and electromagnetism reduce the simpler condition in global Cartesian frame [47]. From the above relation, the following operator form of gauge and potential variables can be found,

| $\chi=-i\bar{\chi}\varphi(r_{i}) ; \chi^{'}=-i\bar{\chi}\varphi^{'}\left( r_{i}^{'} \right) ,$ | (22) |
| --- | --- |

and

| $V=i\bar{\chi}\partial_{t}\varphi; A=-i\bar{\chi}\partial_{r}\varphi\Rightarrow A_{i}=-i\bar{\chi}\partial_{r_{i}}\phi=\bar{A}_{i}\varphi.$ | (23) |
| --- | --- |

### ***Infinitesimal map and minimal coupling***

From the operator form, we further study the matrix transform and connect it with minimal coupling in electrodynamics along geodesics. The variation between actions from Eq. (19) can be connected via phase transform $\Delta\phi$,

| $S^{'}=-i\bar{S}\phi^{'}=-i\bar{S}\phi\cdot\Delta\phi=S\cdot\Delta\phi$ | (24) |
| --- | --- |

and it leads to the following relation of four-momentums $p_{i}$ and $p_{i}^{'}$,

| $p_{i}^{'}=\bar{p}_{i}^{'}\phi^{'}=\bar{p}_{i}^{'}\phi\cdot\Delta\phi=\left( \left[ I_{ii^{'}} \right]+\left[ \sigma_{ii^{'}} \right] \right)p_{i}=\left( \left[ \bar{I}_{ii^{'}} \right]+\left[ \bar{\sigma}_{ii^{'}} \right] \right)\bar{p}_{i}\phi_{\sigma}\cdot\phi$ | (25) |
| --- | --- |

and by equating the phase component, it leads to following phase relation,

| $\phi^{'}=\phi\cdot\Delta\phi=\phi_{\sigma}\cdot\phi.$ | (26) |
| --- | --- |

From the Eq. (4), Eq. (5) and Eq. (28), the infinitesimal map on pointwise characterizations in Eq. (5) can be explicit constructed in matrix-like relation,

| $\left[ \begin{matrix} r_{i}^{'} \\ \bar{p}_{i}^{'}\phi^{'} \\ -i\bar{S}\phi^{'} \end{matrix} \right]=\left[ \begin{matrix} I_{ii^{'}}+\epsilon_{ii^{'}} & & \\ & I_{ii^{'}}+\sigma_{ii^{'}} & \\ & & \Delta\phi(\epsilon) \end{matrix} \right]\left[ \begin{matrix} r_{i} \\ \bar{p}_{i}\phi\\ -i\bar{S}\phi\end{matrix} \right] ,$ | (27) |
| --- | --- |

where the small parameter $\epsilon=\Delta\theta$, and from the exponential map $\Delta\phi\left( \epsilon\right)=I+\epsilon\cong e^{-i\Delta\theta}$. The phase transform $\Delta\phi$ on action is functional dependent on infinitesimal map of coordinates $\Delta r_{i}$and momentum $\Delta\bar{p}_{i}$,

| $\Delta\phi\left( \epsilon\right)=\phi(\Delta\bar{p}_{i},r_{i})\cdot\phi(\bar{p}_{i},\Delta r_{i}) .$ | (28) |
| --- | --- |

The first term reflects the variation from momentum such as the example of minimal coupling between particle and electromagnetic potential in Eq. (8). To see this, we further substitute Eq. (20) and Eq. (23) into Eq. (8), and the following matrix relation can be obtained as,

| $\left[ \sigma_{ii^{'}} \right]p_{i}=\left[ \bar{\sigma}_{ii^{'}} \right]\phi_{\sigma}\bar{p}_{i}\phi=[\bar{q}_{ii^{'}}]\varphi_{q}\bar{A}_{i}\varphi=[q_{ii^{'}}]A_{i}$ | (29) |
| --- | --- |

and through equating the magnitude term and phase terms in Eq. (29), it gives,

| $\left[ \bar{\sigma}_{ii^{'}} \right]\bar{p}_{i}=[\bar{q}_{ii^{'}}]\bar{A}_{i}$ | (30) |
| --- | --- |

and

| $\phi\cdot\Delta\phi=\phi_{\sigma}\cdot\phi=\varphi_{q}\cdot\varphi.$ | (31) |
| --- | --- |

Further by assuming the two matrices $\left[ \bar{\sigma}_{ii^{'}} \right]$ and $[\bar{q}_{ii^{'}}]$ are in diagonal forms, the relation in Eq. (25) becomes to the minimal coupling in classical electrodynamics [48],

| $\Delta\bar{p}_{i}=\bar{p}_{i}^{'}-\bar{p}_{i}=\left[ \bar{\sigma}_{ii^{'}} \right]\bar{p}_{i}=\left[ \bar{q}_{ii^{'}} \right]\bar{A}_{i}=q\bar{A}_{i}$ | (32) |
| --- | --- |

and the phase transform $\Delta\phi$ now has an explicit root of the coupling between four-momentum $\Delta\bar{p}_{i}$ and four-potential $q\bar{A}_{i}$,

| $\Delta\phi=\phi\left( \Delta\bar{p}_{i},r_{i} \right)=\phi\left( \left[ \bar{q}_{ii^{'}} \right]\bar{A}_{i},r_{i} \right)=\phi\left( q\bar{A}_{i},r_{i} \right) .$ | (33) |
| --- | --- |

By applying the results in Eq. (29) and Eq. (32) into Eq. (27), the variation of particle is contributed from the coupling of gauge potential,

| $\left[ \begin{matrix} I_{ii^{'}} & & \\ & \sigma_{ii^{'}} & \\ & & \Delta\phi\end{matrix} \right]\left[ \begin{matrix} r_{i} \\ \bar{p}_{i}\phi\\ -i\bar{S}\phi\end{matrix} \right]=\left[ \begin{matrix} I_{ii^{'}} & & \\ & q_{ii^{'}} & \\ & & \varphi_{q} \end{matrix} \right]\left[ \begin{matrix} r_{i} \\ \bar{A}_{i}\varphi\\ -i\bar{\chi}\varphi\end{matrix} \right]$ | (34) |
| --- | --- |

Moreover, the second term of phase transform in Eq. (28) reflects the variation from the coordinate. This term will be studied in the next section where the relative phase of initial parallel particles is varied from geometry-like potential. A summarized picture of the above results is given in the figure below.


$\bar{S}\to\hbar$ $\phi\to\psi$ $m$ $y_{l}$ $q_{k}=\left[ q_{0}\left( \tau\right),q_{1},q_{2},q_{3} \right]$ $\tau$ $\bar{S}$ $\phi\left( q_{k} \right)$
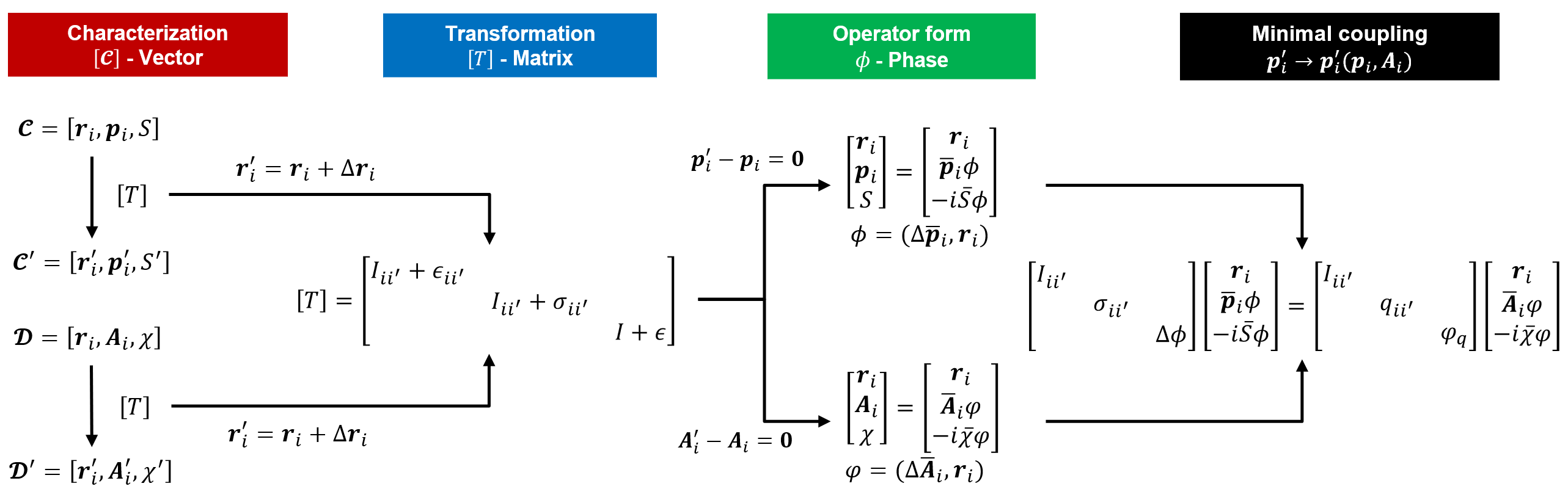


**Figure 1 – Minimal coupling represented from matrix transform of pointwise characterization** $r_{i}$ $q_{k}$ $x_{j}$ $y_{l}$

## Geometric coupling from gravity

In this section, we study the geometric coupling that arise from the geodesic deviation (e.g. tidal force) and its coupling with test particle. Firstly, we study the coordinate deformation from geodesic deviation and establish associated phase transform between initial parallel test particles formally. Secondly, we analyzed connection between field equation and geometric coupling across neighbor geodesics. Subsequently, we study the explicit case for linearized gravitational field and its geometric coupling with test particle.

### ***Geodesics deviation and phase transform***

In differential geometry, the geodesic deviation describes the relative acceleration (four-vector) ${\ddot{\boldsymbol{u}}}_{ib}$ between the two neighbor geodesic curves [9]. Technically, the geodesic deviation comes from non-trivial (Reimann) curvature and represents the differential of the gravitational (tidal) forces between neighbor objects [49]. Firstly, considering the relative four-displacement between the two neighbor geodesics that are initial parallel as the below implicit form,

| $\boldsymbol{y}_{lb}= y_{b}\left( \boldsymbol{x}_{ja},+\boldsymbol{u}_{lb} \right) ; x_{a}= x_{a}\left( y_{b},u_{b} \right) .$ | (35) |
| --- | --- |

and

| $dx_{a}=\nabla_{y_{b}}x_{a}dy_{b}=\left( \nabla_{y_{b}}y_{b}+\nabla_{y_{b}}u_{b} \right)dy_{b}=\varepsilon_{ab}dy_{b} ,$ | (36) |
| --- | --- |


where $\delta_{lj} is the Kronecker delta and \varepsilon_{ab}\varepsilon_{ij}$ is the geodesic strain between neighbor geodesics and it degenerates to identity tensor once two geodesics are in parallel. The geodesic strain is analogous with classical elasticity in tensorial form as in literature [50]. To evaluate the effect of geodesic strain and associated phase transform, we recalled the local coordinates $r_{i}(x_{a})$ and $q_{j}(y_{b})$ along neighbor geodesics. The differential of local inertia coordinates can be represented from covariant derivatives with respect to $x_{a}$ and $y_{b}$,

| $d\boldsymbol{r}_{i}\boldsymbol{=}\nabla_{x_{a}}\nabla_{x}\boldsymbol{r}_{i}dx_{a}x_{j}\boldsymbol{;}d\boldsymbol{q}_{kj}\boldsymbol{=}\nabla_{y_{b}}q_{j}dy_{b}\nabla_{y}q_{i}dy_{l} .$ | (37) |
| --- | --- |

From the substitution of Eq. (36) to Eq. (37), the following relation will arrive,

| $dr_{i}=\nabla_{x_{a}}r_{i}\varepsilon_{ab}dy_{b}=\nabla_{x_{a}}r_{i}\varepsilon_{ab}\left( \nabla_{y_{b}}q_{j} \right)^{-1}=e_{ij}dq_{j}=\left[ \bar{e}_{ij} \right]\varphi_{e}dq_{j} ,$ | (38) |
| --- | --- |


$dx_{j}$ $\epsilon_{ki}$where $\left[ e_{ji} \right]$ refers to matrix that transforms between local inertia coordinates at the two neighbor geodesics, $[\bar{e}_{ji}]$ is the magnitude of coordinate maps (matrix) and $\varphi_{e}$ refers to the phase. $\varepsilon_{lj}$ $\psi\left( r_{i} \right)$ $\psi\left( q_{k} \right)$From complex exponentials in [51] and consider the infinitesimal evolution of the phase functions, it gives,

| $\phi\psi\left( \boldsymbol{r}_{i}+d\boldsymbol{r}_{i} \right)=\phi\psi\left( \boldsymbol{r}_{i} \right)\cdot\phi\psi\left( d\boldsymbol{r}_{i} \right) ; \phi\psi\left( \boldsymbol{q}_{kj}+d\boldsymbol{q}_{kj} \right)=\phi\psi\left( q_{j}q_{k} \right)\cdot\phi\psi\left( dq_{j}q_{k} \right) .$ | (39) |
| --- | --- |

As the two nearby geodesics are initial parallel, one could find two identical local inertia frames that the two test particles carry the same initial states, that is,

| $\bar{p}_{i}k_{i}=\bar{p}_{j}\kappa_{k} ; \boldsymbol{r}_{i}=q_{j}q_{k}\Rightarrow\phi\theta\left( \boldsymbol{r}_{i} \right)=\phi\theta\left( q_{j}q_{k} \right) ,.$ | (40) |
| --- | --- |

$k_{i}$ $\kappa_{k}$During the evolution, the phase functions $\phi\left( dr_{i} \right)$ and $\phi\left( dq_{j} \right)$ will deviate from each other due to the non-identical of $e_{ji}\epsilon_{ki}$,

| $\phi\psi\left( d\boldsymbol{r}_{i} \right)=e^{-i\theta\left( d\boldsymbol{r}_{i} \right)} ; \phi\psi\left( dq_{j}q_{k} \right)=e^{-i\theta\left( dq_{j}q_{k} \right)} ,$ | (41) |
| --- | --- |

where

| $\theta\left( dr_{i} \right)=\frac{1}{\bar{S}}\bar{p}_{i}dr_{i}=\frac{1}{\bar{S}}\bar{p}_{i}\left[ \bar{e}_{ij} \right]dq_{j}=\frac{1}{\bar{S}}\bar{p}_{j}\left[ \bar{e}_{ij} \right]dq_{j} ; \theta\left( dq_{j} \right)=\frac{1}{\bar{S}}\bar{p}_{j}dq_{j} .$ | (42) |
| --- | --- |


By estimating the difference of phase angles between $\theta\left( dr_{i} \right)$ and $\theta\left( dq_{j} \right)$ and noting Eq. (41), it gives,

| $\frac{1}{\bar{S}}\bar{p}_{j}\left[ \bar{e}_{ij} \right]dq_{j}-\frac{1}{\bar{S}}\bar{p}_{j}\left[ I_{ij} \right]dq_{j}=\frac{1}{\bar{S}}\bar{p}_{j}\left[ \bar{f}_{ij} \right]dq_{j}=\frac{1}{\bar{S}} \bar{p}_{i}\left[ \bar{f}_{ij} \right]dq_{j}=\frac{1}{\bar{S}} \bar{p}_{i}\Delta dr_{i}=\frac{1}{\bar{S}}\Delta\bar{p}_{i}dr_{i} ,$ | (43) |
| --- | --- |

where $\left[ \bar{f}_{ij} \right]=\left[ \bar{e}_{ij} \right]-\left[ I_{ij} \right]$ refers to the difference between the matrices, $\Delta dr_{i}=\left[ \bar{f}_{ij} \right]dq_{j}$ refers to variation of coordinate differential and $\Delta\bar{p}_{i}$ refer to the variation of the momentum that keep phase invariance. By substituting the results into Eq. (28), the (relative) phase transform of the test particles at neighbor geodesics due to geometric deformation can be found as

| $\Delta\phi=\phi\left( r_{i}+dr_{i} \right)\cdot{\phi\left( q_{j}+dq_{j} \right)}^{-1}=\phi\left( \bar{p}_{i},\Delta dr_{i} \right)=\phi\left( \Delta\bar{p}_{i},dr_{i} \right)\psi\left( q_{k}+dq_{k} \right)=\psi\left( r_{i}+dr_{i} \right)\cdot\psi_{\epsilon}\left( \theta_{\epsilon} \right) .$ | (44) |
| --- | --- |

By recalling Eq. (28), the above phase transform $\phi\left( \bar{p}_{i},\Delta dr_{i} \right)$ refers to the example of second term where the contribution is from the geometric variations on coordinate. From the point of characterization, the geometric coupling refers the infinitesimal map on coordinate. In this case, the variation of coordinate is independent with gauge coupling that originates from momentum map. At the same time, the inner product between coordinate and momentum (commutative), allows rewrite the phase transform locally as an equivalent geometric-like potential that coupling to the four-momentum $\bar{p}_{i}^{'}\to\bar{p}_{i}+\Delta\bar{p}_{i}$ that leaves coordinate unaltered. Next, we will explore the configuration of geometric coupling from gravitational field equations.


### ***Geometric coupling from gravitational field***

From classical gravity, the (Einstein) field equation defines the connection between Ricci curvature $R_{ljab}$ to the stress-energy tensor $T_{ljab}$ as below [9],

| $R_{ljab}\left( \boldsymbol{x}_{ja} \right)-\frac{1}{2}R\left( \boldsymbol{x}_{ja} \right)g_{ljab}\left( \boldsymbol{x}_{ja} \right)+\Lambda g_{ljab}\left( \boldsymbol{x}_{ja} \right)=\frac{8\pi G}{\boldsymbol{c}^{4}}T_{ljab}\left( \boldsymbol{x}_{ja} \right) ; R\left( \boldsymbol{x}_{ja} \right)=tr\left[ R_{ljab}\left( \boldsymbol{x}_{ja} \right) \right] ,$ | (45) |
| --- | --- |

where $R$ is the scalar curvature (trace of Ricci curvature), $\Lambda$ is the cosmological constant, $g_{ljab}$ is the metric tensor, $G$ is the gravitational constant and Ricci tensor is defined from index contracting of Riemann curvature tensor [52]. Considering the field equation at the point of nearby geodesic denoted by $y_{b}$, the field equation from Eq. (35) remains valid as,

| $R_{ljab}\left( \boldsymbol{y}_{lb} \right)-\frac{1}{2}R\left( y_{b}y_{l} \right)g_{ljab}\left( y_{b}y_{l} \right)+\Lambda g_{ljab}\left( \boldsymbol{y}_{la} \right)=\frac{8\pi G}{\boldsymbol{c}^{4}}T_{ljab}\left( y_{b}y_{l} \right) ; R\left( y_{b}y_{l} \right)=tr\left[ R_{ljab}\left( y_{b}y_{l} \right) \right] .$ | (46) |
| --- | --- |

From Eq. (45) and Eq. (46), the deviations of the nearby metric tensor, Ricci tensor and stress-energy tensor are denoted by,

| $\Delta g_{ljab}=g_{ljab}\left( y_{b}y_{l} \right)-g_{ljab}\left( \boldsymbol{x}_{ja} \right) ; \Delta R_{ljab}=R_{ljab}\left( \boldsymbol{y}_{lb} \right)-R_{ljab}\left( \boldsymbol{x}_{ja} \right) ;\Delta T_{ljab}=T_{ljab}\left( y_{b}y_{l} \right)-T_{ljab}\left( \boldsymbol{x}_{ja} \right) .$ | (47) |
| --- | --- |

By subtracting the Eq. (46) from Eq. (45) and using Eq. (47), the field equation under small variation becomes,

| $\Delta R_{ljab}-\frac{1}{2}\left[ R\left( y_{b}y_{l} \right)g_{ljab}\left( y_{b}y_{l} \right)-R\left( \boldsymbol{x}_{ja} \right)g_{ljab}\left( \boldsymbol{x}_{ja} \right) \right]+\Lambda\Delta g_{ljab}=\frac{8\pi G}{\boldsymbol{c}^{4}}\Delta T_{ljab} ,$ | (48) |
| --- | --- |

where the second term in the bracket can be further expanded with the aid of Eq. (47), it gives,

| $\left[ R\left( \boldsymbol{x}_{ja} \right)+\Delta R \right]\left[ g_{ljab}\left( \boldsymbol{x}_{ja} \right)+\Delta g_{ljab} \right]-R\left( \boldsymbol{x}_{ja} \right)g_{ljab}\left( \boldsymbol{x}_{ja} \right)=R\left( \boldsymbol{x}_{ja} \right)\Delta g_{ljab}+\Delta Rg_{ljab}\left( \boldsymbol{x}_{ja} \right)+\Delta R\Delta g_{ljab} .$ | (49) |
| --- | --- |

Substitution the expansion in Eq. (49) directly into Eq. (48), it becomes,

| $\Delta R_{ljab}-\frac{1}{2}\left[ R\left( \boldsymbol{x}_{ja} \right)\Delta g_{ljab}+\Delta Rg_{ljab}\left( \boldsymbol{x}_{ja} \right)+\Delta R\Delta g_{ljab} \right]+\Lambda\Delta g_{ljab}=\frac{8\pi G}{\boldsymbol{c}^{4}}\Delta T_{ljab} .$ | (50) |
| --- | --- |

Rewritten the above equation with respect to $\Delta g_{ljab}$, it gives,

| $\left[ \Lambda-\frac{1}{2}R\left( \boldsymbol{x}_{ja} \right)-\frac{1}{2}\Delta R \right]\Delta g_{ljab}=\frac{8\pi G}{\boldsymbol{c}^{4}}\Delta T_{ljab}-\Delta R+\frac{1}{2}\Delta Rg_{ljab}\left( \boldsymbol{x}_{ja} \right)$ | (51) |
| --- | --- |

and noting the scalar properties $R$, $\Delta R$ and $\Lambda$, the term $\Delta g_{ljab}$ can be explicitly represented as,

| $\Delta g_{ljab}=\left[ \frac{8\pi G}{\boldsymbol{c}^{4}}\Delta T_{ljab}-\Delta R+\frac{1}{2}\Delta Rg_{ljab} \right]\left[ \Lambda-\frac{1}{2}R-\frac{1}{2}\Delta R \right]^{-1}.$ | (52) |
| --- | --- |

Following the field equation, the above relation reflects the variation of metric tensor along neighboring geodesics. By recalling geometric coupling from neighboring geodesics, the next section will study the connection between geometric coupling and the variation of metric tensor. From definition in differential geometry [53], we consider the small line element of two nearby geodesics,

| $d{s\left( \boldsymbol{x}_{ja} \right)}^{2}=g_{ljab}\left( x_{j} \right)d\boldsymbol{x}^{la}d\boldsymbol{x}^{jb} ; {ds\left( y_{b}y_{l} \right)}^{2}=g_{ljab}\left( y_{l} \right)d\boldsymbol{y}^{la}d\boldsymbol{y}^{jb}$ | (53) |
| --- | --- |

and by using the metric variation in Eq. (53) and the relation in Eq. (36), the variation of the line element can be represented as,

| ${ds\left( y_{b}y_{l} \right)}^{2}-d{s\left( \boldsymbol{x}_{ja} \right)}^{2}=g_{ljab}\left( y_{l} \right)d\boldsymbol{y}^{la}d\boldsymbol{y}^{jb}-g_{ljab}\left( x_{j} \right)d\boldsymbol{x}^{la}d\boldsymbol{x}^{jb}=\left( g_{ab}g_{lj}+\Delta g_{ab}g_{lj} \right)\varepsilon_{ab}\left( \delta_{lj}+\varepsilon_{lj} \right)d\boldsymbol{x}^{la}\varepsilon_{ab}\left( \delta_{lj}+\varepsilon_{lj} \right)d\boldsymbol{x}^{jb}-g_{ab}dx^{a}dx^{b}g_{lj}dx^{l}dx^{j}\boldsymbol{=}\left( g_{ab}\varepsilon_{ab}\varepsilon_{ab}+\Delta g_{ab}\varepsilon_{ab}\varepsilon_{ab}-g_{ab} \right)\left[ \left( g_{lj}+\Delta g_{lj} \right)\left( \delta_{lj}\delta_{lj}+2\delta_{lj}\varepsilon_{lj}+\varepsilon_{lj}\varepsilon_{lj} \right)-g_{lj} \right]d\boldsymbol{x}^{la}d\boldsymbol{x}^{jb}\boldsymbol{.}$ | (54) |
| --- | --- |

By considering the small strain assumption of $\varepsilon_{ab}$ based on the Taylor expansion [49],

| $\varepsilon_{ab}=I_{ab}+\epsilon_{ab}+\frac{1}{2}\epsilon_{ab}^{2}+O(\epsilon_{ab})\cdots\cong I_{ab}+\epsilon_{ab}$ | (55) |
| --- | --- |

and the bracket term in Eq. (54) can be further expanded as,

| $g_{ab}\varepsilon_{ab}\varepsilon_{ab}+\Delta g_{ab}\varepsilon_{ab}\varepsilon_{ab}-g_{ab}\cong\left( g_{lj}+\Delta g_{lj} \right)\left( \delta_{lj}\delta_{lj}+2\delta_{lj}\varepsilon_{lj}+\varepsilon_{lj}\varepsilon_{lj} \right)-g_{lj}=\left( g_{lj}\delta_{lj}\delta_{lj}+2g_{lj}\varepsilon_{lj}+g_{lj}\varepsilon_{lj}\varepsilon_{lj}+\Delta g_{lj}\delta_{lj}\delta_{lj}+2\Delta g_{lj}\delta_{lj}\varepsilon_{lj}+\Delta g_{lj}\varepsilon_{lj}\varepsilon_{lj} \right)\left( g_{ab}I_{ab}I_{ab}+g_{ab}\epsilon_{ab}\epsilon_{ab}+2g_{ab}I_{ab}\epsilon_{ab} \right)+\left( \Delta g_{ab}I_{ab}I_{ab}+\Delta g_{ab}\epsilon_{ab}\epsilon_{ab}+2\Delta g_{ab}I_{ab}\epsilon_{ab} \right)-g_{ab}\cong g_{lj}= 2g_{ljab}\epsilon_{ab}\varepsilon_{lj}+g_{ab}\epsilon_{ab}g_{lj}\varepsilon_{lj}\varepsilon_{lj}\epsilon_{ab}+\Delta g_{ab}\Delta g_{lj}+2\Delta g_{ab}\epsilon_{ab}\Delta g_{lj}\varepsilon_{lj}++\Delta g_{ab}\epsilon_{ab}\epsilon_{ab}\Delta g_{lj}\varepsilon_{lj}\varepsilon_{lj} ,.$ | (56) |
| --- | --- |

If the above second-order small terms including $g_{ab}\epsilon_{ab}\epsilon_{ab}$, $\Delta g_{ab}\epsilon_{ab}$ and higher-order small terms $\Delta g_{ab}\epsilon_{ab}\epsilon_{ab}$ can be neglected from $g_{lj}\varepsilon_{lj}\varepsilon_{lj},$ $\Delta g_{lj}\varepsilon_{lj}$ $\Delta g_{lj}\varepsilon_{lj}\varepsilon_{lj}$the above relation, it gives

| $g_{ab}\varepsilon_{ab}\varepsilon_{ab}+\Delta g_{ab}\varepsilon_{ab}\varepsilon_{ab}-g_{ab}\left( g_{lj}+\Delta g_{lj} \right)\left( \delta_{lj}\delta_{lj}+2\delta_{lj}\varepsilon_{lj}+\varepsilon_{lj}\varepsilon_{lj} \right)-g_{lj}\cong2g_{ab}\epsilon_{ab}2g_{lj}\varepsilon_{lj}+\Delta g_{ab}\Delta g_{lj} .$ | (57) |
| --- | --- |

By substituting the above approximation into Eq. (54), the variation of line element can be found as,

| ${ds\left( y_{b}y_{l} \right)}^{2}-d{s\left( \boldsymbol{x}_{ja} \right)}^{2}\cong\left( 2g_{ab}\epsilon_{ab}+\Delta g_{ab}2g_{lj}\varepsilon_{lj}+\Delta g_{lj} \right)dx^{a}dx^{b}dx^{l}dx^{j}\boldsymbol{.}$ | (58) |
| --- | --- |

and dividing the above equation with reference line element it becomes,

| $\varepsilon\varepsilon_{s}=\frac{{ds\left( y_{b} \right)}^{2}-d{s\left( x_{a} \right)}^{2}{ds\left( y_{l} \right)}^{2}-d{s\left( x_{j} \right)}^{2}}{d{s\left( x_{a} \right)}^{2}d{s\left( x_{j} \right)}^{2}}\cong\frac{\left( 2g_{ab}g_{lj}\epsilon_{ab}\varepsilon_{lj}+\Delta g_{ab}g_{lj} \right)dx^{a}dx^{b}dx^{l}dx^{j}}{g_{ab}dx^{a}dx^{b}g_{lj}dx^{l}dx^{j}}\boldsymbol{=}\frac{2g_{ab}g_{lj}\epsilon_{ab}\varepsilon_{lj}+\Delta g_{ab}g_{lj}}{g_{ab}g_{lj}}\boldsymbol{,}$ | (59) |
| --- | --- |

where $\varepsilon\varepsilon_{s}$ refers to the (scalar) strain of line element. By considering the limit of the $\varepsilon\varepsilon_{s}$ for two neighbor geodesics, the following approximate relation between geodesic strain and metric deviation strain can be obtained,

| $\varepsilon\varepsilon_{s}\cong0 \Rightarrow g_{ab}\epsilon_{ab}\cong g_{lj}\varepsilon_{lj}\cong-\frac{1}{2}\Delta g_{ab}g_{lj} ,$ | (60) |
| --- | --- |

where the variation of metric tensor $\Delta g_{ab}$ from field equation contributes to the infinitesimal geodesic strain $\epsilon_{ab}$. From the relation in Eq. (56), the coordinate map $e_{ij}$ in Eq. (38) can be formally determined from metric tensor,

| $e_{ij}\left( \Delta g_{ab} \right)=\nabla_{\boldsymbol{x}_{a}}\boldsymbol{r}_{i}\varepsilon_{ab}\left( \nabla_{\boldsymbol{y}_{b}}\boldsymbol{q}_{j} \right)^{-1}\cong\nabla_{\boldsymbol{x}_{a}}\boldsymbol{r}_{i}\left[ I_{ab}+\epsilon_{ab} \right]\left( \nabla_{\boldsymbol{y}_{b}}\boldsymbol{q}_{j} \right)^{-1}=\nabla_{\boldsymbol{x}_{a}}\boldsymbol{r}_{i}\left[ I_{ab}-\frac{1}{2}\Delta g_{ab}g_{ab}^{-1} \right]\left( \nabla_{\boldsymbol{y}_{b}}\boldsymbol{q}_{j} \right)^{-1}$ | (61) |
| --- | --- |

and therefore the inner product of phase angle in Eq. (43) becomes,

| $\theta\left( dr_{i} \right)\frac{1}{\bar{S}}\bar{p}_{j}\left[ \bar{e}_{ij} \right]dq_{j}-\frac{1}{\bar{S}}\bar{p}_{j}\left[ I_{ij} \right]dq_{j}=\frac{1}{\bar{S}}\bar{p}_{j}\left[ \bar{f}_{ij} \right]dq_{j}-\theta\left( dp_{j} \right)=\frac{1}{\bar{S}} {\bar{\boldsymbol{p}}}_{i}\Delta d\boldsymbol{r}_{i}\left( \Delta g_{ab} \right)=\frac{1}{\bar{S}}\Delta{\bar{\boldsymbol{p}}}_{i}\left( \Delta g_{ab} \right)d\boldsymbol{r}_{i} .$ | (62) |
| --- | --- |

By substituting the results in Eq. (63) into Eq. (28), the phase transform the coupling of geometric potential in Eq. (44) becomes metric-dependent form,

| $\Delta\phi\phi\left( \bar{p}_{j},dq_{j} \right)\cdot{\phi\left( \bar{p}_{i},dr_{i} \right)}^{-1}==\phi\left( {\bar{\boldsymbol{p}}}_{i},\Delta d\boldsymbol{r}_{i}\left( \Delta g_{ab} \right) \right)=\phi\left( \Delta{\bar{\boldsymbol{p}}}_{i}\left( \Delta g_{ab} \right),d\boldsymbol{r}_{i} \right) .$ | (63) |
| --- | --- |

It is worth to mention that the assumption of infinitesimal strain in Eq. (56) is a simpler case and higher order strain terms are neglected. Also, the approximation between the geodesic strain and variation of metric tensor will incorporate higher order terms if the finite strain assumption is applied. To further study the above formulation on geometric coupling, explicit results will be studied with further condition of linearized field with clear analogy with minimal coupling in gauge-potential field.


### $g_{lj}$ $\eta_{lj}$ $h_{lj}(x_{j})$ $h_{lj}(y_{l})$ $v=c$ $T_{lj}$ $\bar{h}_{lj}$ $\phi_{h}(r_{i}+dr_{i})$ $\phi_{h}(q_{k}+dq_{k})$ $\varepsilon_{lj}$ $\eta^{lj}$ $\Delta\bar{h}_{lj}$ $h_{i}$ $\theta_{\epsilon}$ $\epsilon_{ki}$ $\psi_{\epsilon}$ $\psi$ $\bar{H}$ $\bar{p}$ $\hbar h_{0}$ $\hbar h$***Linearized gravity and minimal coupling***

From the literature of linearized gravity and gravitational waves under harmonic gauge has been well established [54]. The linearized metric tensor $g_{ab}$ is given as the small perturbation from the flat metric,

| $g_{ab}\left( x_{a} \right)=\eta_{ab}+h_{ab}\left( x_{a} \right) ; g_{ab}(y_{b})=\eta_{ab}+h_{ab}(y_{b}) ,$ | (64) |
| --- | --- |

where $h_{ab}(x_{a})$, $h_{ab}(y_{b})$ refer to the small perturbative metrics at nearby geodesics and $\eta_{ab}$ refers to flat metric. Linearized gravitational wave equations on local inertia frames $r_{i}$ and $q_{j}$ at nearby geodesics are given as,

| $\left( \frac{\partial^{2}}{\partial r^{2}}-\frac{1}{c^{2}}\frac{\partial^{2}}{\partial t^{2}} \right)h_{ab}=\frac{16\pi G}{c^{4}}T_{ab}(x_{a}) ; \left( \frac{\partial^{2}}{\partial q^{2}}-\frac{1}{c^{2}}\frac{\partial^{2}}{\partial\tau^{2}} \right)h_{ab}=\frac{16\pi G}{c^{4}}T_{ab}(y_{b})$ | (65) |
| --- | --- |

where the source is governed by stress-energy tensor $T_{ab}$ on the right-hand side. The basis solutions of the above wave equations give,

| $h_{ab}\left( r_{i}+dr_{i} \right)=\bar{h}_{ab}\left( r_{i}+dr_{i} \right)\varphi_{h}\left( r_{i}+dr_{i} \right)$ | (66) |
| --- | --- |

and

| $h_{ab}\left( q_{j}+dq_{j} \right)=\bar{h}_{ab}\left( q_{j}+dq_{j} \right)\varphi_{h}\left( q_{j}+dq_{j} \right) ,$ | (67) |
| --- | --- |

where $\bar{h}_{ab}$ is the magnitude of perturbative metric, $\varphi_{h}(r_{i}+dr_{i})$ and $\varphi_{h}(q_{j}+dq_{j})$ are the phase functions. By applying Eq. (64) into Eq. (57), the deviation of line element under linearized gravity can be found as,

| ${ds\left( y_{b} \right)}^{2}-d{s\left( x_{a} \right)}^{2}\cong\left[ 2\eta_{ab}\epsilon_{ab}+h_{ab}\left( y_{b} \right)-h_{ab}\left( x_{a} \right) \right]dx^{a}dx^{b}$ | (68) |
| --- | --- |

and

| $\Delta g_{ab}\cong h_{ab}\left( y_{b} \right)-h_{ab}\left( x_{a} \right) ,$ | (69) |
| --- | --- |

denotes linearized variation of metric from Eq. (65). With the results in Eq. (54) and Eq. (57), the strain of the line element in Eq. (60) with linearized metric becomes,

| $\varepsilon\cong\frac{2\eta_{ab}\epsilon_{ab}+h_{ab}\left( y_{b} \right)-h_{ab}\left( x_{a} \right)}{\eta_{ab}+h_{ab}\left( x_{a} \right)}$ | (70) |
| --- | --- |

and the original relation in Eq. (60) becomes the follow approximated condition,

| $\varepsilon_{s}\cong0 \Rightarrow\eta_{ab}\epsilon_{ab}\cong-\frac{1}{2}\left[ h_{ab}\left( y_{b} \right)-h_{ab}\left( x_{a} \right) \right] .$ | (71) |
| --- | --- |

Substitution of wave-like solutions from Eq. (66) and Eq. (67) into Eq. (71), the following relations between metric can be found,

| $\epsilon_{ab}\cong-\frac{1}{2}\left[ h_{ab}\left( y_{b} \right)-h_{ab}\left( x_{a} \right) \right]\eta_{ab}^{-1}=-\frac{1}{2}\Delta\bar{h}_{ab}\varphi_{\Delta h}\eta_{ab}^{-1} ,$ | (72) |
| --- | --- |

where $\Delta\bar{h}_{ab}$ refers to relative magnitude and $\varphi_{\Delta h}$ refers to the phase. Under the linearized condition $\epsilon_{ab}$ reduces to a diagonal form (matrix) as flat metric $\eta^{ab}$ is diagonal. With linearized perturbated metric in Eq. (64) and Eq. (72), the coordinate map in Eq. (38) becomes the simplifier form as below,

| $e_{ij}\cong\nabla_{x_{a}}r_{i}\left[ I_{ab}+\epsilon_{ab} \right]\left( \nabla_{y_{b}}q_{j} \right)^{-1}=\nabla_{x_{a}}r_{i}\left[ I_{ab}-\frac{1}{2}\Delta\bar{h}_{ab}\varphi_{\Delta h}\eta_{ab}^{-1} \right]\left( \nabla_{y_{b}}q_{j} \right)^{-1}$ | (73) |
| --- | --- |

and

| $\nabla_{x_{a}}r_{i}\cong\eta_{ia} ; \left( \nabla_{y_{b}}q_{j} \right)^{-1}\cong\eta_{jb}^{-1} ,$ | (74) |
| --- | --- |

where above approximations in Eq. (74) comes from the small perturbation of metrics at nearby geodesics are flat-like. The bracket term in Eq. (73) can be simplified by introducing a relative perturbative (diagonal) tensor $\Delta\bar{h}_{ab}$,

| $e_{ij}\cong\eta_{ia}I_{ab}\eta_{jb}^{-1}-\frac{1}{2}\eta_{ia}\left( \Delta\bar{h}_{ab}\eta_{ab}^{-1} \right)\eta_{jb}^{-1}\varphi_{\Delta h}=I_{ij}-\frac{1}{2}\Delta\bar{h}_{ij}\varphi_{\Delta h}$ | (75) |
| --- | --- |

and

| $\left[ \bar{f}_{ij} \right]=\left[ \bar{e}_{ij} \right]-\left[ I_{ij} \right]=-\frac{1}{2}\Delta\bar{h}_{ij} ,$ | (76) |
| --- | --- |

where $\Delta\bar{h}_{ji}$ refers to the magnitude of coordinate map under linearized condition. Then by substituting Eq. (76) into phase in Eq. (43) and noting the initial condition in Eq. (40), it gives,

| $\theta\left( dr_{i} \right)-\theta\left( dp_{j} \right)=\frac{1}{\bar{S}}\bar{p}_{i}\left[ \bar{f}_{ij} \right]dq_{j}\cong-\frac{1}{\bar{S}}\bar{p}_{i}\frac{1}{2}\Delta\bar{h}_{ij}dq_{j}=\frac{1}{\bar{S}}\bar{p}_{i}\Delta dr_{i}=\frac{1}{\bar{S}}\bar{h}_{i}dr_{i}$ | (77) |
| --- | --- |

where $\bar{h}_{i}$ refers to the variation of momentum-like variables that unalter the phase angle. By substituting the results in Eq. (63) into Eq. (28), the specific phase transform $\Delta\phi$ from the coupling of geometric potential via gravitational field effect can be formally found below,

| $\Delta\phi=\phi\left( \bar{p}_{i},\Delta dr_{i} \right)=\phi\left( \bar{h}_{i},dr_{i} \right) .$ | (78) |
| --- | --- |

To visualize the above relations in this section, a diagram is presented in Fig. 2 to describe the key procedures. Additionally, by comparing the results in Eq. (77) and Eq. (78), it manifests formal analogies with gauge coupling in Eq. (33). In the former case, the gauge coupling contributes to phase via transformation of the four-momentum by four-potential $\bar{A}_{i}$. In the latter case, the geometric coupling (under linearized condition) contributes to the phase via transformation of the coordinate via tensorial map. By fulfilling the invariance of phase, the commutative of inner product allows to introduce an equivalent four-potential denoted by $\bar{h}_{i}$ to transform the four-momentum from the geometric coupling. From the operator form, the four-potential $\bar{A}_{i}$ and $\bar{h}_{i}$ are locally analogous with each other that subjected to the same coupling scheme, as represented below

| $\Delta\phi(\bar{A}_{i})=\phi\left( \bar{A}_{i},\Delta dr_{i} \right) \sim\phi\left( \bar{h}_{i},dr_{i} \right)=\Delta\phi(\bar{h}_{i}) .$ | (79) |
| --- | --- |


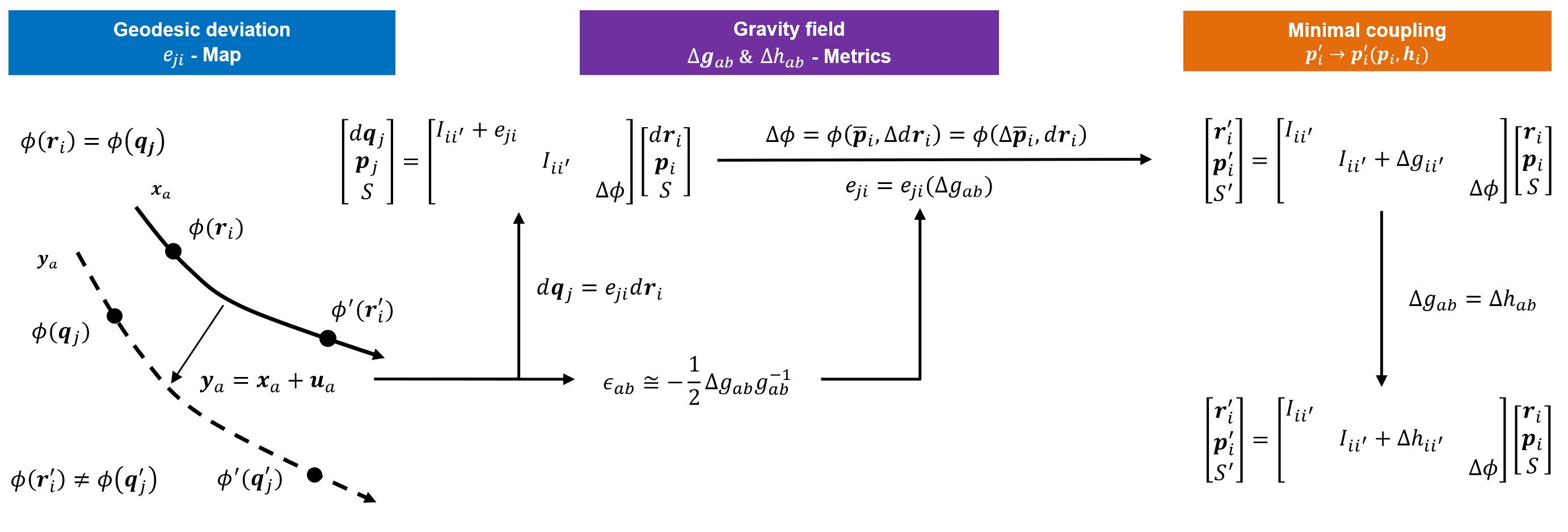


**Figure 2 – Geometric coupling as matrix transforms from general and linearized gravitational field**


## Quantum formulation and application

From the view of isomorphism, we study the quantum form of particle and potentials through functional maps. Subsequently, the equivalent principle is discussed based on the coupling between gauge coupling and geometric coupling to reveal the analogies. Finally, some potential quantum applications of geometric coupling in gravitational field are discussed.

### ***Isomorphism and quantum map***

In abstract (modern) algebra, isomorphism has been raised from different branches that represent the one-to-one relation between the structures of the two objects [55 - 57]. From the operator form of particle along geodesics and by comparing the theoretical structures, we notice the formal isomorphism between the operator form and quantum form on action and dynamic variables. More specifically, the original operator form is structural invariance under the function mapping of amplitude $\bar{S}$ and phase $\phi$. Firstly, considering the following function mapping $F$,

| $\hbar=F\left( \bar{S} \right)\Rightarrow F\left( S \right)=-iF\left( \bar{S} \right)\phi\left( r_{i} \right)=-i\hbar\phi\left( r_{i} \right).$ | (80) |
| --- | --- |

As both $\bar{S}$ and $\hbar$ (Planck constant) are action-like time-independent, the mapping represents a scaling $F=\lambda$ in the real domain. Secondly, considering the following function mapping $H$ between the phases,

| $\psi\left( r_{i} \right)=H\left[ \phi\left( r_{i} \right) \right]\Rightarrow H\left[ S \right]=-i\hbar H\left[ \phi\left( r_{i} \right) \right]=-i\hbar\psi\left( r_{i} \right) .$ | (81) |
| --- | --- |

As $\phi\left( r_{i} \right)$ and $\psi\left( r_{i} \right)$ are dimensionless complex exponentials, the mapping connects both phase via global change $H\sim e^{-i\alpha}$ in complex domain [50]. From the pointwise characterization in Eq. (5), the set of function mappings $F$ and $H$ in Eq. (80) and Eq. (81), provide an isomorphic relation between original operator form and quantum form on a pointwise basis upon geodesics. It degenerate to the simpler form as the coordinate is global Cartesian frame [58]. It is worth to note that there is no derivable argument to specify the translation of amplitude $\bar{S}$ and phase $\phi$ into corresponding quantized form. Therefore, the isomorphism relation only reflects the formal invariance of the theoretical structures of multiple analogous objects without completely specify. Thus, the original operator form becomes,

| $p_{i}\left( \bar{S},\phi\right)\Rightarrow p_{i}\left( \hbar,\psi\right)=-i\hbar\partial_{r_{i}}\psi=\bar{p}_{i}\psi=\hbar k_{i}\psi$ | (82) |
| --- | --- |

and the resultant dynamic operator is consistent with quantum formulation. Furthermore, the minimal coupling between particle and gauge potential given in Eq. (33) can be represented as,

| $\Delta\phi\Rightarrow\Delta\psi=\Delta\psi\left( q\bar{A}_{i},r_{i} \right) .$ | (83) |
| --- | --- |

The minimal coupling between particle and geometric-like potential in Eq. (78) can be represented as,

| $\Delta\phi\Rightarrow\Delta\psi=\Delta\psi\left( \bar{h}_{i},dr_{i} \right) .$ | (84) |
| --- | --- |

By substituting the above relations into the infinitesimal map of characterization in Eq. (27), the matrix relation becomes,

| $\left[ \begin{matrix} r_{i}^{'} \\ \bar{p}_{i}^{'}\psi^{'} \\ -i\hbar\psi^{'} \end{matrix} \right]=\left[ \begin{matrix} I_{ii^{'}} & & \\ & I_{ii^{'}}+q_{ii^{'}}+\Delta h_{ii^{'}} & \\ & & \Delta\psi\left( \bar{A}_{i},\bar{h}_{i} \right) \end{matrix} \right]\left[ \begin{matrix} r_{i} \\ \bar{p}_{i}\psi\\ -i\hbar\psi\end{matrix} \right]$ | (85) |
| --- | --- |

where $\Delta h_{ii^{'}}$ refers to the coupling of geometric potential, $q_{ii^{'}}$ refers to the coupling of gauge potential and $\Delta\psi$ reflects the resultant phase transform that can be represented from Eq. (83) and Eq. (84) as,

| $\Delta\phi\Rightarrow\Delta\psi=\psi\left( q\bar{A}_{i},r_{i} \right)\cdot\psi\left( \bar{h}_{i},dr_{i} \right) .$ | (86) |
| --- | --- |

At the same time, the magnitude of the gauge potential and geometric potential can be resolved from the quantized momentum as,

| $\Delta\bar{p}_{i}=\hbar k_{i}^{'}-\hbar k_{i}=q\bar{A}_{i}+\bar{h}_{i}$ | (87) |
| --- | --- |

By imposing the above potential terms into operator in Eq. (82), the following Schrödinger-like equation of particle under field potentials can be found with energy-momentum relation [7],

| $i\hbar\partial_{t}\psi=\left[ \frac{1}{2m}\left( -i\hbar\partial_{r}-q\bar{A}-\bar{h} \right)^{2}+q\bar{V}+\bar{h} \right]\psi.$ | (88) |
| --- | --- |

where $\bar{A}$ and $\bar{h}$ denote the vectorial-like potentials, $V$ and $h$ denote the scalar-like potentials. As one of the special cases, the above equation is back to the free particle form when both $\bar{A}_{i}\to0$ and $\bar{h}_{i}\to0$ terms become negligible. The relativistic version of the above equation can be obtained via imposing relativistic energy-momentum relation [59].

### ***Local equivalence and phase transform***

According to equivalent principle [60 - 62], the effect of gravitational field and external field are allowed to have cancelled each other locally. By considering the equivalent condition for gauge and geometric potentials, then it provides an equality condition on both phase and magnitude on the coupling potentials. For the infinitesimal map of characterization Eq. (85), the effect of equivalent principle represents an identical map. This indicates the inverse relation on phase transforms from gauge coupling and geometric coupling,

| $-i\hbar\psi^{'}=-i\hbar\psi\cdot\Delta\psi\left( \bar{A}_{i},\bar{h}_{i} \right)=-i\hbar\psi$ | (89) |
| --- | --- |

and from Eq. (86) it can be further expanded as below,

| $\Delta\psi=\psi\left( q\bar{A}_{i},dr_{i} \right)\cdot\psi\left( \bar{h}_{i},dr_{i} \right)=I\Rightarrow\psi\left( q\bar{A}_{i},dr_{i} \right)={\psi\left( \bar{h}_{i},dr_{i} \right)}^{-1} .$ | (90) |
| --- | --- |

By expanding each term and removing common term $dr_{i}$ from the phase, it leads to the below condition to fulfill local equivalence,

| $\left( q\bar{A}_{i}-\bar{h}_{i} \right)dr_{i}=0\Rightarrow q\bar{A}_{i}-\bar{h}_{i}=0$ | (91) |
| --- | --- |

and

| $\bar{h}=-q\bar{V} ; \bar{h}=-q\bar{A} .$ | (92) |
| --- | --- |

Based on the local equivalence, the above relation bridges the equality between the gauge potential and geometric potential. By applying the above condition, $q\bar{A}=-\hbar\bar{h}$ and $q\bar{V}=-\hbar\bar{h}$, into operators in Eq. (86), the governing equation reduces to,

| $i\hbar\partial_{t}\psi=\left[ \frac{1}{2m}\left( -i\hbar\partial_{r}-\left( q\bar{A}+\bar{h} \right) \right)^{2}+\left( q\bar{V}-\bar{h} \right) \right]\psi\Rightarrow i\hbar\partial_{t}\psi=\frac{-\hbar^{2}}{2m}\partial_{r}^{2}\psi,$ | (93) |
| --- | --- |

where this gives another special case of free-condition form with non-trivial gauge potentials and geometric potentials. In classical field theories, the force balance at a local point is typically treated from the magnitude aspect, whereas in the operator form, the local equivalence is also constrained by the phase function, as in Eq. (89). The explicit condition in Eq. (91) that comes from the equivalent principle shows the local analogies between the gauge coupling and geometric coupling upon the particle in operator form. In addition to analogous formulations, this result has practical implications for applications. For instance, detectable changes in the energy–momentum through variations in the wave parameters (e.g., frequency and wavelength) owing to the geometric coupling can be predicted. In addition, the phase shift owing to the interference effect from the geometric potential and particle wavefunction can be predicted, similar to cases in electromagnetism.

### ***Gravitational shift and potential well***


$h_{i}$ $A_{i}$Gravitational red shift is known as the geometric effect of particle travel away from potential well. From geometric coupling, we consider a simplified case as in Newtonian gravity [54],

| $U\left( d \right)=\frac{GMm}{d} ; F=-mg,$ | (94) |
| --- | --- |

where $M$ denotes mass of the object, $m$ denotes mass of the test particle, $d$ denotes the relative distance, $U$ refer to the field potential, $G$ is the potential force and $g$ is the gravitational acceleration on the earth. The following expression of scalar potential can be obtained,

| $h=E=mU.$ | (95) |
| --- | --- |

and the expression of vector potential can be obtained

| $\bar{h}=\int Gdt=-mgt .$ | (96) |
| --- | --- |

By substituting the above expression into Eq. (85) and using wave parameters,

| $\Delta\bar{H}=\bar{h}=\hbar\omega^{'}-\hbar\omega; \Delta\bar{p}=\bar{h}=\hbar k^{'}-\hbar k$ | (97) |
| --- | --- |

it gives the correlation between gravitational field parameters with variation of angular frequency $\omega$ and wave length $\lambda=2\pi/k$through the geometric coupling. The earlier experiment of frequency shift of particle move away from gravitational field has been reported in earlier and recent literature [63 - 65].

Moreover, for a finite four-potential well in a given boundary, the permissible condition of quantized momentum is given by,

| $L_{i}=\frac{n}{2}\lambda_{i}=n\frac{\pi}{k_{i}} \Rightarrow p_{i}=n\frac{\pi\hbar}{L_{i}} ,$ | (98) |
| --- | --- |

where $n$ is the order number of eigenmode of solution. The relation in Eq. (98) leads to the discretization of geometric coupling in gravitational field between the $m$-th order and $n$-th order,

| $\Delta p_{i}=n\frac{\pi\hbar}{L_{i}}-m\frac{\pi\hbar}{L_{i}}=\frac{\pi\hbar}{L_{i}}\left( n-m \right)=\frac{1}{n}p_{i}$ | (99) |
| --- | --- |

and from Eq. (77) and Eq. (78) it has,

| $\Delta\bar{p}_{i}=\frac{1}{n}\bar{p}_{i}=\frac{\Delta\bar{h}_{ij}dq_{j}}{dr_{i}}\bar{p}_{i}=\frac{\Delta dr_{i}}{dr_{i}}\bar{p}_{i}\Rightarrow\Delta\bar{h}_{ij}dq_{j}=\frac{1}{n}dr_{i}$ | (100) |
| --- | --- |

and thus

| $\left[ \bar{e}_{ij} \right]=I_{ij}-\frac{1}{2}\Delta\bar{h}_{ij}=I_{ij}\left( 1-\frac{1}{2n} \right) .$ | (101) |
| --- | --- |

The coordinate map $\bar{e}_{ji}(n)$ becomes discretized as function of order number. By substituting above relation into Eq. (38) and noting the arbitrariness of covariant terms $\nabla_{y_{a}}q_{j}$ and $\nabla_{x_{b}}r_{i}$, it suggests that the geodesic strain $\varepsilon_{ab}$ in Eq. (56) has the following discretized form,

| $e_{ji}\cong\nabla_{y_{a}}q_{j}\left[ I_{ab}+\epsilon_{ab} \right]\left( \nabla_{x_{b}}r_{i} \right)^{-1}\Rightarrow\varepsilon_{ab}=\varepsilon_{ab}\left( 1+\frac{1}{2n} \right) .$ | (102) |
| --- | --- |

The above results indicate the discretized condition can be possible found in the gravitational field that is similar with knew problem in finite potential well in literature. Nevertheless, different from the electrical case where scalar potential is discretized, the results suggests that the discretization of neighbor geodesics (geometric) by the allowable order of eigenmodes. In returns, this implies the geometric configuration is discontinued with certain gap-band between the adjacent geodesics.

### ***Geometric Aharonov–Bohm effect***

According to literature [66], the phase difference of Aharonov–Bohm effect in electromagnetism is depending on the $\boldsymbol{A}_{i}$ on quantum particles. In electromagnetism, Aharonov–Bohm effect is an important test to support the phase dependency of gauge coupling between momentum and potentials [67]. By studying equivalent principle and revealed analogies between gauge coupling and geometric coupling, one might expect a similar phase shift effect from geometric coupling in gravitational potential. As in electromagnetism, the expression the phase difference from vector potential and scalar potential can be found as,

| $\Delta\psi\varphi\left( AA \right)=\frac{q}{\hbar}\Phi_{B}=\frac{q}{\hbar}\oint\boldsymbol{A}dl ; \Delta\psi\left( V \right)=\frac{q}{\hbar}\oint Vdt ,$ | (103) |
| --- | --- |

where $l$refers to certain closed path. By substituting Eq. (92) into above relation, the created phase difference due to geometric effect in gravitational field can be represented as,

| $h=-\frac{q}{\hbar}A\Rightarrow\Delta\psi\varphi\left( hA \right)=\frac{q}{\hbar}\oint Adl=\oint\boldsymbol{h}dl=\Delta\varphi\left( h \right) .$ | (104) |
| --- | --- |


and the scalar case,

| $h=-\frac{q}{\hbar}V\Rightarrow\Delta\psi\left( h \right)=\int hdt .$ | (105) |
| --- | --- |

A similar form of phase shift as in Eq. (102) has been experimentally observed in literature with advanced cold atom devices [68]. More detailed information of the experimental approach and system has been reported in the literature [69]. Recent study on laser interferometer also observe the phase change effect from gravitational waves [70]. In general, the phase difference from gravitational potentials $\boldsymbol{h}_{i}$ can be found as the superposition of the two individual cases as below,

| $\Delta\psi\left( h_{i} \right)=\Delta\varphi\left( h_{i} \right)=\Delta\varphi\left( h,h_{0} \right)=\oint\boldsymbol{h}dl+\int h_{0}hdt .$ | (106) |
| --- | --- |

From the view of isomorphism, the introduced function maps explicit connect the action-like parameter (amplitude) and exponential function (phase) from the original operator form with quantum formulation. Since the function maps do not alter the theoretical structure of the operator form, the forward or inverse application of the function maps will lead to results in similar theoretical form without specified the quantized amplitude and phase.


$U$ $M$ $m$ $d$

# Discussion

In this article, we studied the geometric potential in gravitational field and its coupling with quantum particles as summarized in Fig. 3. In the previous subsection, pointwise characterization of particles and fields is introduced to analyze the evolution of test particles. It is locally given as a vector-like object that allows matrix transformations between different configurations. Geometrically, the pointwise characterization can be considered as (vector-like) characterization spaces from four-displacement $r_{i}$and four-momentum $p_{i}$where action is a governing scalar. Technically, the pointwise feature allows the explicit construction of certain (theoretical) structures without assuming the global properties of the domain (e.g., spacetime). This represents a relatively weaker condition compared with the domain-wise feature (e.g., a stronger condition). In this case, the operator form is pointwise established along the infinitesimal line element of the geodesics. Intuitively, the operator form along the geodesics is like a moving frame that travels along the curves with certain conditions holding [71, 72].

By introducing pointwise characterizations of particles as vector-like objects, we connect that the operator form from an identical map on four-momentum. From the operator form, the coupling of particles from different potentials can be represented as an infinitesimal transform on characterizations, leading to two types of coupling. The former refers to the coupling between the gauge potential (for example, electromagnetism) and momentum, which is transformed from the charge matrix of the four potentials. The latter is studied in more detail and arises from the geometric deformation of the coordinates, which is related to the gravitational force (for example, the tidal effect). In this type, the coordinate map is the key process that contributes to the phase transform in the operators. The coordinate map, metric, and stress–energy tensors are connected using the field equation of gravity. This provides a formal relation for the phase transform from the metric and stress–energy tensors.

During the study of the geometric potential, established concepts concerning the deformation and strain tensor in classical elasticity are incorporated. Although the original elastic tensor is given in three-dimensional space, the concept can be extended into the four-vector form. Specifically, the small strain-like approximation is adapted to simplify the high-order small terms in the metric representation. Technically, the finite strain-like formulation can be incorporated into the aforementioned procedures, wherein the high-order terms between the geodesic strain and metric tensor are counted. Nevertheless, for the linearized field condition, the small strain approximation is sufficient to describe the geometric potential. Subsequently, from the observed isomorphism between the operator and quantum forms, a set of functional maps that retain the structural invariance of the operator form is utilized. Technically, the function maps translate the amplitude and phase in the original operator form into quantum counterparts. It is noted that the amplitude and phase are not specified from the isomorphism, whereas the isomorphism only preserves the results in a formal analogous under different function maps.


$\left\{ r_{i},p_{i},S \right\}$With the above, we could classify the two types of coupling schemes via diagram upon the neighbor geodesic lines, as in Fig. 3. To achieve this, transform matrices can be rewritten by the above studied field variables such as,

| $\left[ \begin{matrix} \boldsymbol{\vdots} \\ \mathcal{C} \\ \boldsymbol{\vdots} \end{matrix} \right]\boldsymbol{=}\left[ \begin{matrix} \cdots& & \\ & T & \\ & & \cdots\end{matrix} \right]\left[ \begin{matrix} \boldsymbol{\vdots} \\ \mathcal{C}^{'} \\ \boldsymbol{\vdots} \end{matrix} \right]\Rightarrow\left[ T \right]=\left[ \begin{matrix} I_{ii^{'}}+\epsilon_{ii^{'}}\left( \Delta g_{ab} \right) & & \\ & I_{ii^{'}}+\sigma_{ii^{'}}\left( q_{ii^{'}} \right) & \\ & & I+\epsilon\left( \Delta\theta\right) \end{matrix} \right] ,$ | (107) |
| --- | --- |

where the infinitesimal transform becomes trivial evolution once $\epsilon_{ii^{'}}$, $\sigma_{ii^{'}}$ and $\epsilon$ become null that $[T]$ becomes uniformly identity. The above couplings are also encoded in the phase transform $\Delta\psi$ as the last main diagonal element,

| $\epsilon=-i\Delta\theta;\Delta\theta=\Delta\theta\left( \left[ \epsilon_{ii^{'}} \right]\boldsymbol{p}_{i},\left[ q_{ii^{'}} \right]\boldsymbol{A}_{i} \right) ; I+\epsilon=e^{-i\Delta\theta}=\Delta\psi.$ | (108) |
| --- | --- |

The pointwise characterizations are denoted by red dots, and the transforms are denoted by arrows with two colors. Each color represents one type of coupling that has a unique matrix representation, and their composition can also be expressed in matrix form. Along each geodesic (for example, left and right), the gauge-like couplings transform the characterization without explicitly altering the coordinate. In the classical field, there are multiple gauge-like couplings that manifest isomorphism with electromagnetism [73, 74]. Across the neighboring geodesics (for example, up and down), the geometric-like couplings transform the coordinate and induce a local equivalent gauge-like potential from the inner product. From a geometric perspective, because geometric coupling is governed by the strain or curvature, general relativity and its approximated versions or similar will manifest geometric coupling by altering the strain or curvature [75].


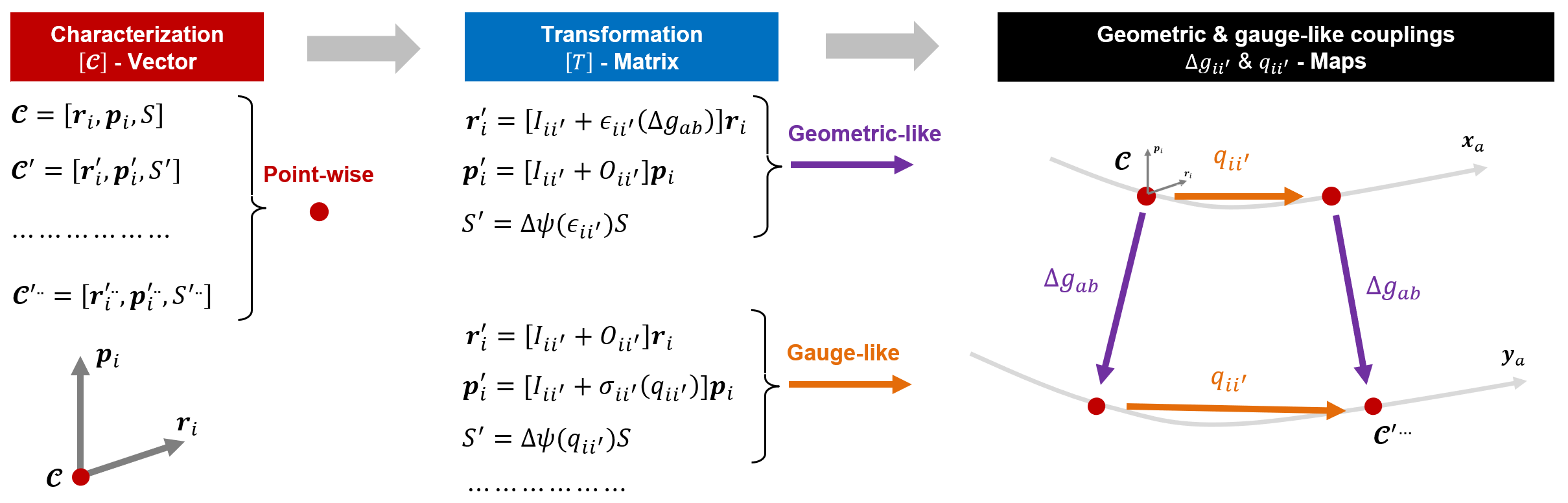


**Figure 3 – Diagram of two coupling schemes from pointwise characterization and matrix transform**

# Methods

## Metric and Christoffel symbol

In differential geometry [53], the Christoffel symbol is defined as the local connections between the two curvilinear frames as below:

| $\Gamma_{jk}^{i}\boldsymbol{e}_{i}=\frac{\partial}{\partial\boldsymbol{r}_{k}}\boldsymbol{e}_{j} ,$ | (109) |
| --- | --- |

where subscript $i$ denotes the index and $\Gamma_{jk}^{i}$ is the Christoffel symbol of the second kind in terms of metric tensor as,

| $\Gamma_{jk}^{i}=\frac{1}{2}g_{im}\left( \frac{\partial}{\partial\boldsymbol{r}_{k}}g_{im}+\frac{\partial}{\partial\boldsymbol{r}_{k}}g_{mk}-\frac{\partial}{\partial\boldsymbol{r}_{m}}g_{kj} \right) .,$ | (110) |
| --- | --- |

where $g_{im}$ is the metric tensor. From the Christoffel symbol in Eq. (92), the Riemann curvature tensor can be obtained as,

| $R_{jkl}^{i}=\Gamma_{jk}^{m}\Gamma_{ml}^{i}-\Gamma_{jl}^{m}\Gamma_{mk}^{i}+\frac{\partial}{\partial\boldsymbol{r}_{l}}\Gamma_{jk}^{i}-\frac{\partial}{\partial\boldsymbol{r}_{k}}\Gamma_{jl}^{i} .$ | (111) |
| --- | --- |

## Small and finite strain form

In classical elasticity or classical field theories [50], the displacement field gradient (tensor) of the medium at smooth domain can be given as the rigid-body (reference) displacement and elastic deformation,

| $F_{ij}=\frac{\partial}{\partial x_{j}}\left( X_{i}+u_{i} \right)=\frac{\partial X_{i}}{\partial x_{j}}+\frac{\partial u_{i}}{\partial x_{j}}=\delta_{ij}+\varepsilon_{ij} ,$ | (112) |
| --- | --- |

where $X_{i}$refers to the reference displacement, $u_{i}$refers to the deformation upon the coordinate $x_{j}$and $\varepsilon_{ij}$ refers to the elastic strain tensor. In the usual notation, the coordinate is configured as Cartesian frame with three-dimensional space. By expansion of the strain tensor, the (Cauchy) small strain formulation is given as,

| $\varepsilon_{ij}=\frac{1}{2}\left( \frac{\partial u_{i}}{\partial x_{j}}+\frac{\partial u_{j}}{\partial x_{i}} \right) ,$ | (113) |
| --- | --- |

where the differential is taken up to linear order. The (Green-Lagrange) finite strain formulation is given by

| $E_{ij}=\varepsilon_{ij}+\frac{1}{2}\left( \frac{\partial^{2}u_{i}}{\partial x_{j}^{2}} \right)+\cdots+O\left( dx_{j} \right) ,$ | (114) |
| --- | --- |

where $O(dx_{j})$refers to the high-order small terms.

# References

1. Lanczos C. The variational principles of mechanics. Courier Corporation; 2012.
2. Holm DD, Schmah T, Stoica C. Geometric mechanics and symmetry: from finite to infinite dimensions. Oxford University Press; 2009.
3. Hertz H, Jones DE. The principles of mechanics presented in a new form. Macmillian and Company, Limited; 1899.
4. Lützen J. Mechanistic Images in Geometric Form: Heinrich Hertz's' Principles of Mechanics'. OUP Oxford; 2005.
5. Einstein A. The general theory of relativity. In The meaning of relativity 1922 (pp. 54-75). Dordrecht: Springer Netherlands.
6. Ehlers J. Survey of general relativity theory. In Relativity, Astrophysics and Cosmology: Proceedings of the Summer School Held, 1972 at the Banff Centre, Banff, Alberta 1973 (pp. 1-125). Dordrecht: Springer Netherlands.
7. Messiah A. Quantum mechanics. Courier Corporation; 2014.
8. Peskin ME, Schroeder DV. An Introduction to. Quantum Field Theory. 1995:69-76
9. Weinberg S. Gravitation and cosmology: principles and applications of the general theory of relativity. John Wiley & Sons; 2013.
10. Jackson JD, Okun LB. Historical roots of gauge invariance. Reviews of modern physics. 2001;73(3):663.
11. Carlip S. Quantum gravity: a progress report. Reports on progress in physics. 2001;64(8):885.
12. Kiefer C. Quantum gravity: general introduction and recent developments. Annalen der Physik. 2006 12;518(1-2):129-48.
13. Callender C, Huggett N, editors. Physics meets philosophy at the Planck scale: Contemporary theories in quantum gravity. Cambridge University Press; 2001.
14. Smolin L. Three roads to quantum gravity. Basic books; 2008.
15. Abbott LF. The background field method beyond one loop. Nuclear Physics B. 1981;185(1):189-203.
16. Ivanenko D, Sardanashvily G. The gauge treatment of gravity. Physics Reports. 1983;94(1):1-45.
17. Vilkovisky GA. Effective action in quantum gravity. Classical and Quantum Gravity. 1992;9(4):895.
18. Tod P, Moroz IM. An analytical approach to the Schrödinger-Newton equations. Nonlinearity. 1999;12(2):201.
19. Ashtekar A, Lewandowski J. Background independent quantum gravity: A status report. Classical and Quantum Gravity. 2004;21(15):R53.
20. Percacci R. An introduction to covariant quantum gravity and asymptotic safety. World Scientific; 2017 .
21. Ashtekar A, Bianchi E. A short review of loop quantum gravity. Reports on Progress in Physics. 2021;84(4):042001.
22. Singh TP, Padmanabhan T. Notes on semiclassical gravity. Annals of Physics. 1989;196(2):296-344.
23. Kiefer C. The semiclassical approximation to quantum gravity. InCanonical Gravity: From Classical to Quantum: Proceedings of the 117th WE Heraeus Seminar Held at Bad Honnef, Germany, 13–17 September 1993 2005 Jul 24 (pp. 170-212). Berlin, Heidelberg: Springer Berlin Heidelberg.
24. Shapere A, Wilczek F, editors. Geometric phases in physics. World scientific; 1989.
25. Berry MV. Evolution of semiclassical quantum states in phase space. Journal of Physics A: Mathematical and General. 1979;12(5):625-42.
26. Berry MV. Semi-classical mechanics in phase space: a study of Wigner’s function. Philosophical Transactions of the Royal Society of London. Series A, Mathematical and Physical Sciences. 1977 Oct 4;287(1343):237-71.
27. Wands D. Extended gravity theories and the Einstein--Hilbert action. Classical and Quantum Gravity. 1994;11(1):269.
28. Sotiriou TP, Faraoni V. f (R) theories of gravity. Reviews of Modern Physics. 2010;82(1):451-97.
29. Capozziello S, De Laurentis M. Extended theories of gravity. Physics Reports. 2011;509(4-5):167-321.
30. Capozziello S, Francaviglia M. Extended theories of gravity and their cosmological and astrophysical applications. General Relativity and Gravitation. 2008;40:357-420.
31. Capozziello S, D’Agostino R, Luongo O. Extended gravity cosmography. International Journal of Modern Physics D. 2019;28(10):1930016.
32. Wallace D. Quantum gravity at low energies. Studies in History and Philosophy of Science. 2022;94:31-46.
33. Shankaranarayanan S, Johnson JP. Modified theories of gravity: Why, how and what?. General Relativity and Gravitation. 2022;54(5):44.
34. Anastopoulos C, Hu BL. Problems with the Newton–Schrödinger equations. New Journal of Physics. 2014;16(8):085007.
35. Isham CJ. Prima facie questions in quantum gravity. In Canonical Gravity: From Classical to Quantum: Proceedings of the 117th WE Heraeus Seminar Held at Bad Honnef, Germany, 13–17 2005 (pp. 1-21). Berlin, Heidelberg: Springer Berlin Heidelberg.
36. Kiefer C. Why quantum gravity?. In Approaches to Fundamental Physics: An Assessment of Current Theoretical Ideas 2007 (pp. 123-130). Berlin, Heidelberg: Springer Berlin Heidelberg.
37. Carlip S. Is quantum gravity necessary?. Classical and Quantum Gravity. 2008;25(15):154010.
38. Törmä P. Essay: Where can quantum geometry lead us?. Physical Review Letters. 2023;131(24):240001
39. Sorkin RD. Forks in the road, on the way to quantum gravity. International Journal of Theoretical Physics. 1997;36:2759-81.
40. Terno DR. Inconsistency of quantum--classical dynamics, and what it implies. arXiv preprint quant-ph/0402092. 2004.
41. Borzeszkowski HH, Treder HJ. The meaning of quantum gravity. Springer Science & Business Media; 2012.
42. Loll R, Fabiano G, Frattulillo D, Wagner F. Quantum gravity in 30 questions. arXiv preprint arXiv:2206.06762. 2022.
43. Crowther K. Why Do We Want a Theory of Quantum Gravity?. Cambridge University Press; 2025
44. Cullen CG. Matrices and linear transformations. Courier Corporation; 2012.
45. Yang C. Analogy between electromagnetic potentials and wave-like dynamic variables with connections to quantum theory. European Journal of Physics. 2018 39(3):035406.
46. Jackson JD. Classical electrodynamics, 3rd edition. Wiley; 1998;
47. Wald RM. General relativity. University of Chicago press; 2010.
48. McConnell AJ. Applications of tensor analysis. Courier Corporation.
49. Lovelock D, Rund H. Tensors, differential forms, and variational principles. Courier Corporation; 1989.
50. Gilmore R. Lie groups, Lie algebras, and some of their applications. Courier Corporation; 2006.
51. McWeeny R. Symmetry: An introduction to group theory and its applications. Courier Corporation; 2012.
52. Landau LD, editor. The classical theory of fields. Elsevier; 2013.
53. Pound RV, Rebka Jr GA. Gravitational red-shift in nuclear resonance. Physical Review Letters. 1959;3(9):439.
54. Turneaure JP, Will CM, Farrell BF, Mattison EM, Vessot RF. Test of the principle of equivalence by a null gravitational red-shift experiment. Physical Review D. 1983;27(8):1705.
55. Bothwell T, Kennedy CJ, Aeppli A, Kedar D, Robinson JM, Oelker E, Staron A, Ye J. Resolving the gravitational redshift across a millimetre-scale atomic sample. Nature. 2022 ;602(7897):420-4.
56. Batelaan H, Tonomura A. The Aharonov–Bohm effects: Variations on a subtle theme. Physics Today. 2009;62(9):38-43.
57. Peshkin M. The Aharonov-Bohm effect: Why it cannot be eliminated from quantum mechanics. Physics Reports. 1981;80(6):375-86.
58. Abramovici A, Althouse WE, Drever RW, Gürsel Y, Kawamura S, Raab FJ, Shoemaker D, Sievers L, Spero RE, Thorne KS, Vogt RE. LIGO: The laser interferometer gravitational-wave observatory. science. 1992 Apr 17;256(5055):325-33.
59. Brading K, Castellani E, editors. Symmetries in physics: Philosophical reflections. Cambridge University Press; 2003. Olver PJ. An introduction to moving frames. InProceedings of the Fifth International Conference on Geometry, Integrability and Quantization 2004 (Vol. 5, pp. 67-81). Bulgarian Academy of Sciences, Institute for Nuclear Research and Nuclear Energy.
60. Olver PJ. Moving frames. Journal of Symbolic Computation. 2003;36(3-4):501-12.
61. Yang C. Quantization of linear acoustic and elastic wave models in characterizations of isomorphism. Scientific Reports. 2024;14(1):8759.
62. Yang C. Quantization of nonequilibrium heat transport models based on isomorphism and gauge symmetry. Scientific Reports. 2025;15(1):1-3.
63. Poisson E, Will CM. Gravity: Newtonian, post-newtonian, relativistic. Cambridge University Press; 2014.

# Funding Statement:

The authors (s) received no financial support for the research, authorship, or publication of this article.

# Conflict of Interest declaration:

The authors declare that they have no affiliations with or involvement in any organization or entity with any financial interest in the subject matter or materials discussed in this manuscript.

# Data availability:

All data generated or analyzed during this study are included in this published article.

1. Authors email address (primary): [chen.yang@manchester.ac.uk](file:///C:\Users\cheny\AppData\Local\Temp\OneNote\16.0\Exported\%7BC7BE5DDA-39A4-4A34-BE97-DBD5A9ACC85B%7D\NT\1\chen.yang@manchester.ac.uk); [↑](#footnote-ref-1)
